# Anti-inflammatory, Analgesic and Antiulcer properties of *Porphyra vietnamensis*

**Published:** 2015

**Authors:** Saurabh Bhatia, Kiran Sharma, Ajay Sharma, Kalpana Nagpal, Tanmoy Bera

**Affiliations:** 1 *PDM College of Pharmacy, Bahadurgah, Haryana, India.*; 2 *Deptt. of Pharmaceutical Sciences, Jamia Hamdard, New Delhi, India.*; 3 *Seoul National University, Republic of Korea, Korea.*; 4 *Division of Pharmaceutics, Faculty of Medical Sciences, Lovely Professional University, Phagwara, Punjab India.*; 5 *Department of Pharmaceutical Technology,Jadavpur University, Kolkata, India*

**Keywords:** *Porphyra*, *Porphyran*, *Carrageenan*, *Analgesia*, *Ulcer*

## Abstract

**Objectives:** Aim of the present work was to investigate the anti-inflammatory, analgesic and antiulcer effects of red seaweed Porphyra vietnamensis (P. vietnamenis).

**Materials and Methods:** Aqueous (POR) and alcoholic (PE) fractions were successfully isolated from P. vietnamenis. Further biological investigations were performed using a classic test of paw edema induced by carrageenan, writhing induced by acetic acid, hot plate method and naproxen induced gastro-duodenal ulcer.

**Results**: Among the fractions POR showed better activity. POR and PE significantly (p < 0.05) reduced carrageenan induced paw edema in a dose dependent manner. In the writhing test POR significantly (p < 0.05) reduced abdominal writhes than PE. In hot plate method POR showed better analgesic activity than PE. POR showed comparable ulcers reducing potential (p<0.01) to that of omeprazole, and has more ulcer reducing potential then PE.

**Conclusions**: The results of this study demonstrated that P. vietnamenis aqueous fraction possesses biological activity that is close to the standards taken for the treatment of peripheral painful or/and inflammatory and ulcer conditions.

## Introduction

Inflammation is the protective host defense mechanism of the body characterized by redness, heating, swelling, pain and loss of function of the affected area. Inflammation aids disposal of microorganisms and helps to restore tissue homeostasis. Inflammation that occurs in the mucosal of gastrointestinal tract, however, causes gastrointestinal ulcer (Wakefield and Kumar, 2001 ▶). Gastrointestinal ulcer is a common major disorder of the digestive system affecting millions of peoples (Sonnenberg, 1994 ▶). Inhibition of the activity of inflammatory cells or inhibition of the production of inflammatory mediators is known to be the general strategies for their treatment. Nonsteroidal anti-inflammatory drugs (NSAID) and steroidal anti-inflammatory drugs (SAID) are widely used in the treatment of these diseases. Gastric discomfort, nausea and vomiting are the most common side effects caused by aspirin or indomethacin (Tripathi 2003 ▶). In addition, anti-inflammation and anti-ulcerogenic drugs are used mainly to alleviate the symptoms of the disease without actually treating or preventing the inflammatory and ulcerogenic processes. Nevertheless, there are many natural products that exhibit anti-inflammatory and analgesic properties and have relatively low incidences of side effects. Various studies on marine organisms are demonstrating that many compounds produced by marine life have useful pharmacological activities (Mayer and Lehmann, 1998 ▶). The marine environment is a rich source of both biological and chemical diversity. In recent years, a significant number of novel metabolites with potent pharmacological properties have been discovered from the marine organisms. Among these organisms, the seaweeds or macroalgae are considered to be a rich source of bioactive compounds suitable for therapeutic and medical applications. Emerging trend of increasing new molecules from macroalgae promotes the marine science towards the potential research area of drug discovery. Commercially available varieties of marine macroalgae are commonly referred to as ‘seaweeds” (Rinehart et al., 1998 ▶). Seaweeds are the futuristically promising plants, act as one of the most important marine resources. These plants can be used as source of food, feed and medicine from ancient times. Although, seaweeds in India are used for industrial production of agar and alginate and as a fertilizer medicinal use of sea weed has not yet been explored. Being a plant of unique structure and biochemical composition, seaweed could be explored for various purposes in the form of food, medicine and cosmetics (DeVries et al., 1995; De Vries and Beart, 1995; Faulkner, 1995 ▶; Carté, 1996 ▶; Haefner, 2003 ▶). 

Production of safe and potent analgesic, anti-inflammatory and anti-ulcerogenic drugs from natural origin has recently been focused. Due to wide abundance algae could be a potential source of new therapeutic compounds (Smit, 2004 ▶; Dhargalkar and Pereira, 2005 ▶). The red algae has been reported to contain active compounds that may help amelioration of inflammation of the alimentary tract (Kang et al., 2008 ▶), prevention and treatment of gastric ulcers and cancers caused by oxidative stress, inflammatory activities by suppressing the production of inflammatory mediators and induction of cancer cell apoptosis in stomach and colon (Khan et al., 2008 ▶; Gonzalez et al., 2009 ▶). Natural compounds derived from the edible algae would be safer to be used as anti-inflammatory and gastric anti-ulcerogenic therapeutics as they were taken as food and used in traditional medicines since time immemorial (Dhargalkar and Pereira, 2005 ▶; Shu et al., 2013 ▶). 

*Porphyra,* popularly known as ‘Nori’ in Japan, ‘Kim’ in Korea and ‘Zicai’ in China has an annual value of over US $ 1.8 billion. By considering its traditional uses it is very essential to evaluate the nutritional and physiological functions of its constituents. *Porphyra* (Bangiales, Rhodophyta) is a good taste traditional medicinal food which is widely consumed in East Asia. In our previous work we had reviewed the biological activities of *Porphyra *sp. and its cell wall polysaccharide porphyran (Bhatia et al., 2008; Bhatia et al., 2010a, b; Bhatia et al., 2011 ▶; Bhatia et al., 2013 ▶). It is evident that algae produce many important biologically-active compounds, some of which might one day find here used as novel drugs. Thus, there is a need to explore the chemical profile of this alga to bring its medicinal components from the sea to commercial market.

**Experimental section**

**Algal material**

*Porphyra vietnamensis *was collected from the different coastal regions of Maharastra, India. Further taxonomic identification was conducted and a voucher specimen has been deposited in the herbarium at the Laboratory of Ecology under the voucher specimen number (Bot/571/09).

**Preparation of extracts **To isolate the POR and PE from *Porphyra* two different procedures have been adopted. To extract alcoholic fraction soxhlet extraction method was adopted (Bhatia el., 2010a; Bhatia et al., 2013 ▶). For soxhlet method five grams of dried algae was powdered and extracted with a mixture of methanol : water (80:20, v/v), followed by CH_2_Cl_2_. The extract was lyophilized till dryness, to yield 2.1 g of a pale yellow powder. To extract the POR cold extraction method was adopted. In cold extraction method 5 g of dried algal material was dissolved in 250 ml of distilled water and kept in orbital shaking incubator for 12 h at 20-25°C degree. Insoluble fraction was removed by centrifugation (15, 000 rpm at 4°C), supernatant was separated and treated with ethanol (1:3 v/v). Ethanol precipitated fraction was again dissolved in distilled water and dialyzed. The obtained dialyzed sample was lyophilized to obtain 0.38 g product (Bhatia el., 2010a; Bhatia et al., 2013 ▶).

**Animals and treatments**

Swiss albino mice (25-35 g) and male wistar rats (200-230 gm) were of either sex divided into 4 groups (I – IV): Group I (Control) received normal saline; Group II, Porphyran (POR) 250 mg/kg body weight; Group III, *Porphyra* extract (PE) 200 mg/kg; and Group IV, Standard drug of theoretical dose selected for each model and each group comprised of a six animals. Four groups of animals were selected for each study. Animals were preferred according to the previous reports, sensitivity of the model and suitable doses designed for each study. Wistar rats (200-230 gm) were selected for naproxen induced ulcer and carrageenan induced paw edema models whereas swiss albino mice were experimented for rest of the models. For one week before the experiment, the animals were kept in a room at 22 °C with artificial 12:12 h light: dark cycle in ventilated plastic cages. Animals were fed with a standard rodent diet and sterile water was supplied *ad libitum. *Animals were randomized into treatment groups. The experimental protocols were approved by the Institutional Animal Ethics Committee of the PDM College of pharmacy, Haryana, India. Experiments were conducted in accordance with the internationally accepted principles for laboratory animal use and care (PDM/CPCSEA/RES/2012/01).

**Carrageenan induced paw edema in rats**

 This method described by Winter et al., (1962) ▶ was used for evaluating anti-inflammatory activity. Edema was induced by subcutaneous administration of 0.1ml of 1% aqueous solution of carrageenan into right hind paw. Different fractions of *Porphyra *sp. in 1% solution of CMC (caboxy methyl cellulose) and diluted with normal saline were given as oral dose. Wistar rats were divided into four groups (n=6). Group I, received normal saline (10 ml/kg body wt.); Group II, POR (250 mg/kg body wt); Group III, PE (250 mg/kg body wt.); and Group IV, received indomethacin (reference drug 10 mg/kg). Carrageenan paw volume was measured up to 6 hr after the carrageenan administration at an interval of 60 min by plethysmometer (Guillen et al., 1997 ▶) .

**Acetic acid induced writhing response**

 It was evaluated using the method of Whittle (1999) ▶. Swiss albino mice were divided into four groups (n=6). Sterile saline (control group, 0.9%, w/v), indomethacin (10 mg/kg) and test solutions (POR 250 mg/kg & PE 250 mg/kg) were administered orally 30 min before the experiment and 0.7% acetic acid (10 mL kg^−^^1^ body weight) was then injected i.p. 10 min after the injection. The number of writhing during the following 20 min period was counted. The percent inhibition (% analgesic activity) was calculated by 

$\mathrm{\% Inhibition}=\frac{N-N^{t}}{N}\times 100$

Where, N = Average number of stretching of control per group 

 N^t^ = Average number of stretching of test per group. 

**Hot plate method**

 The device consisted of a water bath in which a metallic cylinder was placed. Swiss albino mice were divided into four groups (n = 6). The temperature of the cylinder was set at 550.5^o^C (Lanhers et al., 1991 ▶). The reaction time following the administration of the extract (POR 250 mg/kg & PE 250 mg/kg), indomethacin (10 mg/kg) and distilled water (10 ml/kg) was measured at 0 hour to 2-hour after a latency period of one hour. 

**Naproxen induced ulcer**

Control was administered with 1% CMC (caboxy methy cellulose). Control, test drugs (PE & POR at 250 mg/kg) and omeprazole (30 mg/kg p.o.) were administered to four groups (n=6) of Wistar rats. One hour later naproxen 30 mg/kg p.o. was administered. Animals were sacrificed after six hours of naproxen administration, stomach was isolated and opened along greater curvature to expose inner surface. Inner surface was washed thoroughly with normal saline. The ulcer area, ulcer index and the percentage inhibition were calculated as described above (Kim et al., 2005 ▶).

**Statistical analyses**

The results were expressed as mean (±) standard deviation of mean (SD). Analysis of variance (ANOVA) was done to compare and analyze the data followed by Duncan’s multiple range test. Effects were considered significant at p < 0.05. 

**Result and discussion**

**Carrageenan induced paw edema in rats**

POR and PE extract significantly and dose dependently reduced carrageenan induced paw edema compared to control in rats at 6^th^ hr. The standard indomethacin showed better inhibitory activity than fractions of *Porphyra sp.* The percentage decrease in paw volume determines anti-inflammatory potential of the drug*. * The inhibitory activity of the POR is very close to indomethacin. After 24^th ^hr of drug administration POR showed maximum suppression of inflammation. Among all fractions POR showed maximum anti-inflammatory activity (Table1). 

**Acetic acid induced writhing response**

The different fractions significantly reduced acetic acid induced writhing response. Among the fractions POR showed best activity. POR was proved to be more active in inhibition than *Porphyra *extract (Table 2). 

**Table 1:** Effect of *Porphyra *fractions on carrageenan induced inflammation

**Drug**	**% of Inhibition of paw edema** **Responses ** **after treatment time (hrs)**				
	**1 hr**	**3 hr**	**6 hr**	**24 hr**	**% IE**
**Cg control **	1.9±0.011	3.7±0.024	5.2±0.028	4.2±0.024	-
** PE**	1.5±0.015	1.8±0.024	1.7±0.024	1.6±0.024	69.22
** POR**	1.4±0.012	1.6±0.032*	1.5±0.014	1.4±0.027*	75.49
**Indomethacin**	1.4±0.023	1.6±0.029**	1.3±0.017	1.1±0.024**	84.21

Value represent the mean ± S.E.M; Cg (carrageenan), POR (porphyran), PE (*Porphyra *alcoholic extract), % IE (percentage inhibition of edema);

*: *p*<0.01,

**: *p*<0.005 dunnet test.

**Table 2:** Effects of *Porphyra* and its fractions on writhing response

**Drug**	**Response**	**% inhibition**
**AA Control**	51.2±0.124	0
**PE**	28.3±0.112	44.7
**POR**	18.1±0.024*	61.8
**Indomethacin**	9.51.96**	72.7

Value represent the mean ± S.E.M; POR (porphyran), AA (acetic acid), PE (*Porphyra *alcoholic extract);

*: *p*<0.01,

**: *p*<0.005 dunnet test.

**Table 3:** Effects of Porphyra on Hot plate response.

** Drug**	**Reaction time**			
	**0 min**	**30 min**	**60 min**	**90 min**
**Control**	1.7±0.013	1.8±0.012	2.0±0.016	2.5±0.031
**PE**	2.1±0.022	3.0±0.014	3.6±0.011	7.03±0.001
**POR**	2.4±0.014	4.7±0.021 **	5.6±0.021*	11.4±0.014**
**Indomethacin**	3.4±0.021	4.1±0.028**	7.3±0.018**	13.1±0.031**

Values are presented as mean ± SEM, (n=6),

*: *p*<0.01,

**: *p*<0.005 dunnet test as compared to control; POR (porphyran), PE (*Porphyra *alcoholic extract)

**Table 4:** Antiulcer activity of different samples *Porphyra* Sp.

**Treatment**	**Dose**	**US (mm** ^2^ **)**	**UI**	**% I**
**Control 1% CMC**	1ml/animal	5.11 ± 0.122	1.11 ± 0.02	-
**Omiprazole**	30mg/kg	2.16 ± 0.010**	0.41 ± 0.04	57.19
**POR **	250 mg/kg	2.91 ± 0.013*	0.57 ± 0.01	49.03
**PE**	250 mg/kg	4.82 ± 0.015	0.89 ± 0.03	17.01

Value represent the mean ± S.E.M. of gastric lesion area the result was analyzed by ANOVA followed by Dunnetts multiple comparison test

*: *p*<0.01,

**: *p*<0.005, CMC (carboxy methyl cellulose), POR (porphyran), PE (*Porphyra *alcoholic extract) and, US: ulcer surface, UI: ulcer index, % I: percent inhibition

**Hot plate method**

The different fractions at a dose 250 mg/kg show comparable effect than indomethacin 10 mg/kg (Table 3). The extracts of both the algae fractions were found to exhibit a dose dependent increase in latency time when compared with control. At 90 minutes, responses at dose (250 mg/kg body weight) were 7.03 & 11.4 for PE and POR respectively. The results were found to be statistically significant (p<0.001). In the present study two samples of *Porphyra* were studied for their antiulcer activity through naproxen ulcer induced model (Table 4).

**Naproxen induced ulcer**

POR (49.03%) showed much ulcers reducing potential in comparison to omeprazole (57.19%) as mentioned in Table 4. Furthermore it was observed that POR (49.03%) has more potency in reducing ulcer in comparison to PE (17.01%).

## Discussion

The anti-inflammatory and analgesic activities of different fractions of* Porphyra sp.* were investigated in the present study. The carrageenan test was selected because of its sensitivity in detecting orally active anti-inflammatory agents particularly in the acute phase of inflammation. First phase of action of carrageenan results from the concomitant release of mediators: histamine, serotonin and kinins those induces vascular permeability. The second phase is correlated with leukotrienes. The oral administration of different fractions of PE and POR suppresses inflammation during the second phase. 

The POR (250 mg/kg) showed maximum inhibitory response in comparison to PE. The mechanism for testing analgesics was selected in such a way that both centrally and peripherally mediated effects could be investigated. The acetic acid induced abdominal constriction while the hot plate method elucidated peripheral effects (Suzuki et al., 2012 ▶; Yoshida et al., 2002). 

PE and POR (250 mg/kg) administered orally, significantly inhibited acetic acid induced writhing in rats. Their writhing is related to increase in the peritoneal level of prostaglandins and leukotrienes. The result strongly suggests that the mechanism of action of drug may be linked to lipoxygenase and/or cycloxygenase (Suzuki et al., 2012 ▶; Yoshida et al., 2002). . 

The hot plate method has been found to be suitable for evaluation of centrally acting analgesics (Woolfe and MacDonald, 1994 ▶). The nociceptors seem to be sensitized by sensory nerves. The involvement of endogenous substances such as prostaglandins may be minimized in this model. In centrally acting analgesic methods, the drug in 250 mg/kg dose was found to be almost effective than standard indomethacin.

Gastric hyperacidity and gastro-duodenal ulcer is a very common global problem today. Hyperacidity (hyperchlorhydria) is a pathological condition due to uncontrolled hypersecretion of hydrochloric acid from the parietal cell of the gastric mucosa through the proton pump. Hyper-secretion of acid aggravates gastro-duodenal ulcer caused by loss of gastro-protection by various factors. Stress-related gastric mucosal damage, Helicobacter pylori infection and non-steroidal anti-inflammatory drug (NSAID) use are well established risk factors for gastrointestinal mucosal injury (Suzuki et al., 2012 ▶; Yoshida et al., 2002). Apart from the damaging role of acid, reactive oxygen species (ROS) especially the hydroxyl radical (.OH) plays a major role in causing oxidative damage of mucosa in almost all types of ulcer (Suzuki et al., 2012 ▶; Yoshida et al., 2002). Currently a concept is emerging that gastric mucosal lesions caused by various factors are due to increased cell death by apoptosis with simultaneous block of cell proliferation process (Suzuki et al., 2012 ▶; Yoshida et al., 2002). 

Stress induced gastric erosions have been shown to be associated with not only a decrease in gastric blood flow and lowering of proliferating cell nuclear antigen labeling index, but it accompanies also with an imbalance between Bcl-2 family of antiapoptotic protein and Bax protein which promotes apoptosis (Qiao et al., 2011 ▶). Ethanol and indomethacin (NSAID) cause gastric mucosal damage by apoptosis through induction of tumor necrosis factor-α (TNF-α) and DNA damage (Slomiany et al., 1997 ▶).

Indomethacin induced apoptosis also involves release of cytochrome from mitochondria to cytosol and subsequent activation of caspase-3 like protease to cause cell death. Indomethacin also augments gastric lesions by delaying the ulcer healing process through inhibition of angiogenesis by its direct effect on the endothelial cell (Bandyopadhyay et al., 2002 ▶). *Helicobacter pylori* induce gastric epithelial cell apoptosis by its lipopolysaccharide and *urease* or by nitric oxide production through induction of nitric oxide *synthase* and DNA fragmentation. Role of ROS on DNA damage and apoptosis has also been reported (Dirk et al., 2001 ▶).

The modern approach to control gastro-duodenal ulcer has therefore been suggested to inhibit gastric acid secretion, to improve gastro-protection, to scavenge ROS and to eradicate *Helicobacter pylori *(Bandyopadhyay et al., 2002 ▶). Although H_2_-receptor blockers (ranitidine, famotidine), proton-pump inhibitors (omeprazole, lansoprazole), antibiotics (metronidazole, amoxicillin, clarithromycin, tetracycline etc.) and other drugs are currently used for the efficient management of the peptic ulcer disease, they have some limitations also (Pandit et al., 2008 ▶).

Among the natural sources the red algal components have a complex mechanism which requires attention by which they are providing protection against gastric ulcers. Among these algae *Porphyra* contains several prominent components which have a remarkable effect mainly by controlling *H. pylori* growth (Lee et al., 2013 ▶).

Further chemical analysis on the composition *P. vietnamenis *aqueous and alcoholic fractions necessary to isolate and identify bioactive compounds that may have applications in therapeutic fields of inflammation and pain.
